# Supplementary material for: MetaRibo-Seq measures translation in microbiomes
Source: Nat Commun. 2020 Jun 29;11:3268. doi: 10.1038/s41467-020-17081-z (PMC7324362; doi:10.1038/s41467-020-17081-z)
Supplement: Supplementary file 10 — Supplementary Data 7 [file 41467_2020_17081_MOESM10_ESM.zip › File2/Confidence_High_Taxonomy/292272_out.krona.html]

Javascript must be enabled to view this page.

members
magnitude
magnitudeUnassigned
count
unassigned
taxon
rank

292272\_out

4

4
2
superkingdom

976
4
phylum

class
4
200643

order
171549
4

family
815
4

genus
816
4

species

SRS013800\_contig\_number\_17772SRS017821\_contig\_number\_contig-100\_697.195597SRS054352\_contig\_number\_19631SRS893383\_contig\_number\_8925
4
28111
